# Electro-Acupuncture Promotes the Differentiation of Endogenous Neural Stem Cells via Exosomal microRNA 146b After Ischemic Stroke

**DOI:** 10.3389/fncel.2020.00223

**Published:** 2020-07-21

**Authors:** Shenghang Zhang, Tingting Jin, Lulu Wang, Weilin Liu, Yuhao Zhang, Yi Zheng, Yunjiao Lin, Minguang Yang, Xiaojun He, Huawei Lin, Lidian Chen, Jing Tao

**Affiliations:** ^1^College of Rehabilitation Medicine, Fujian University of Traditional Chinese Medicine, Fuzhou, China; ^2^The 900 Hospital of the Joint Logistic Team, Fuzhou, China; ^3^Fujian University of Traditional Chinese Medicine, The Academy of Rehabilitation Industry, Fuzhou, China

**Keywords:** electro-acupuncture, ischemic stroke, neural stem cells, exosomes, MiR-146b

## Abstract

**Background:** Evidences indicate that exosomes-mediated delivery of microRNAs (miRNAs or miRs) is involved in the neurogenesis of stroke. This study was to investigate the role of exosomal miRNAs in non-drug therapy of electro-acupuncture (EA) regulating endogenous neural stem cells for stroke recovery.

**Methods:** The model of focal cerebral ischemia and reperfusion in rats were established by middle cerebral artery occlusion (MCAO) and treated by EA. The exosomes were extracted from peri-ischemic striatum and identified by exosomal biomarkers, and detected differentially expressed miRNAs with microarray chip. Primary stem cells were cultured, and oxygen–glucose deprivation and reperfusion (OGD/R) was used to mimic vitro ischemic injury.

**Results:** The levels of exosomal biomarkers TSG101 and CD81 were increased in peri-ischemic striatum after EA treatment, and we revealed 25 differentially expressed miRNAs in isolated exosomes, of which miR-146b was selected for further analysis, and demonstrated that EA increased miR-146b expression and its inhibitors could block the effects. Subsequently, we confirmed that EA upregulated miR-146b expression to promote neural stem cells differentiation into neurons in peri-ischemic striatum. *In vitro*, it was verified that OGD/R hindered neural stem cells differentiation, and miR-146b inhibitors furtherly suppressed its differentiation, simultaneously NeuroD1 was involved in neural stem cells differentiation into neurons. Moreover, *in vivo* we found EA promoted NeuroD1-mediated neural stem cells differentiation via miR-146b. In addition, EA also could improve neurological deficits through miR-146b after ischemic stroke.

**Conclusion:** EA promotes the differentiation of endogenous neural stem cells via exosomal miR-146b to improve neurological injury after ischemic stroke.

## Introduction

Stroke remains the second-leading cause of death in the world, with ischemic stroke accounting for ~70% of all strokes, and approximately half of patients died or became disabled within 1–3 months (Wang et al., 2017; Wu et al., 2019). Although extensive advance on the epidemiology, etiology, mechanism, classification, and prognosis of stroke, safe and effective treatment strategies have not been developed for most patients, which seriously threatens human health and life, and bring huge burden to patients and families. Therefore, it is an important issue to comprehensively understand the severity of stroke, improve treatment methods, reduce the incidence, disability, and mortality of stroke, and find safe and effective treatment for stroke patients.

Studies have shown that cells of the adult mammalian central nervous system have destructive repair capabilities, especially the proliferation and differentiation of endogenous neural stem cells that can promote neurogenesis (Shinozuka et al., 2014; Nakagomi et al., 2019). The endogenous neural stem cells of the adult brain are mainly present in the subventricular zone (SVZ) region of the lateral ventricle and the subgranular zone (SGZ) region of the hippocampus (Péron and Berninger, 2015; Boldrini et al., 2018). These neural stem cells could be activated and proliferated after stroke then migrated into the lesion and differentiated into functional neural cells (Yamashita et al., 2006; Ngalula et al., 2015). However, newly generated neural stem cells and neural cells by self-healing are not sufficient to promote neurological recovery after stroke. Therefore, how to promote the proliferation and differentiation of endogenous neural stem cells may be a potentially beneficial treatment for stroke.

The previous study has proved that low-frequency electro-acupuncture (EA) stimulation can promote the proliferation of endogenous neural stem cells in SVZ and hippocampus of rats with focal cerebral ischemia (Guo et al., 2014). It has been reported that proliferated endogenous neural stem cells were migrated from the SVZ region to the damaged region of the striatum after EA treatment (Yang et al., 2005). While in our previous study, we demonstrated that EA could promote the proliferation and differentiation of endogenous neural stem cells, which improved the neurological deficits, thereby saving the brain from ischemic damage (Tao et al., 2014; Chen et al., 2015). However, its mechanism is not completely clear.

As we know, secretory extracellular vesicles (EVs) play an essential role in intercellular signal transduction. Recent studies suggest that exosomes-mediated delivery of microRNAs (miRNAs or miRs) is involved in the neurogenesis of stroke (Zagrean et al., 2018; Geng et al., 2019). Exosomes, as a type of EVs (about 30–100 nm in diameter), target cellular functions by delivering proteins, lipids, and nucleic acids (Mori et al., 2019). They can almost be released by all known cell types, including neurons, astrocytes, etc. (Takeda et al., 2015). In this study, we found that exosomal miR-146b identified by microarray chips affected the differentiation of endogenous neural stem cells in rats with ischemia-reperfusion injury. The miR-146 family (miR-146a and miR-146b) has been proved that it can promote the differentiation of neural stem cells into neurons by regulating the Notch1 signaling pathway (Xiao et al., 2015). As a downstream mediator of Notch1 signaling, NeuroD1 is specifically expressed in developing neurons and it is a key transcription factor regulating the differentiation and maturation of neurons (Beckervordersandforth et al., 2015). NeuroD1 transcription factor induced new neuronal cells in the ipsilateral cerebral cortex and lateral striatum of mouse brain after stroke (Gonçalves et al., 2016). Studies have shown that a single transcription factor NeuroD1 in the mouse brain directly converts reactive astrocytes into functional neurons (Guo et al., 2014). And in their recent study, ectopic expression of NeuroD1 in the ischemic injury model through the adeno-associated virus system could regenerate 30–40% of neurons damaged in the motor cortex, and behavioral tests showed that NeuroD1 treatment significantly relieves motor and fear memory deficits after rodent ischemic injury (Chen et al., 2020). Besides, our previous studies have shown that EA could regulate the Notch1 signal transduction to promote the proliferation and differentiation of neural stem cells after ischemic stroke (Tao et al., 2014; Zhao et al., 2015). Therefore, we aimed to demonstrate whether EA could promote the differentiation of endogenous neural stem cells via exosomal miR-146b regulating the NeuroD1 after ischemic stroke.

## Materials and Methods

### Animals

Sixty healthy SPF male *SD* rats (260 ± 20 g) obtained from the Shanghai Laboratory Animal (SLAC, Co., Ltd., Shanghai, China), license no. SCXK 2014−007. All animal experiments were carried out in a pathogen-free environment at the Animal Experimental Center of Fujian University of Traditional Chinese Medicine, placing the rats in a controlled environment (22–25°C; 50 ± 10% relative humidity; 12 h automatic light/dark cycle).

### Experimental Design

This study was divided into two parts. First, to explore which exosomal miRNAs were regulated by EA treatment in rats with ischemic stroke. Rats were divided into three groups (*n* = 7/group): (i) sham operation group (Sham), (ii) middle cerebral artery occlusion group (MCAO), (iii) middle cerebral artery occlusion with EA treatment group (MCAO+EA). Second, to clarify the function of EA treatment effect miRNAs expression in rats with ischemic stroke. Rats were divided into four groups (*n* = 10/group): (i) MCAO group (MCAO), (ii) MCAO and miR-146b inhibitor injection group (MCAO+miR-146b inhibitors), (iii) MCAO and EA treatment group (MCAO+EA), (iV) MCAO and EA treatment combined with miR-146b inhibitors injection group (MCAO+EA+miR-146b inhibitors). The EA treatment continued for 21 days after the operation (1/20 Hz, 1 mA, 30 min/day). The EA needle was inserted into the LI11 and ST36 of hemiplegic limb at a depth of 2–3 mm and stimulation generated with an EA instrument (G6805; SMIF, Shanghai, China). The MCAO+miR-146b inhibitors group and the MCAO+EA+miR-146b inhibitors group were injected with miR-146b inhibitor in the intraventricular 30 min before modeling. The rats were anesthetized with 3% isoflurane (R510, RWD Life Science Co., Ltd., Shenzhen China) and placed on a stereotaxic instrument (68001; RWD Life Science Co., Ltd., Shenzhen China). Stereotactic coordinates were as follows: Anteroposterior, 0.8 mm; Mediolateral, 1.5 mm; Depth, 3.5 mm.

### Focal Cerebral Ischemia Model

We used thread occlusion of the right middle cerebral artery (MCAO) to establish a rat model of focal cerebral ischemia and reperfusion according to the Longa EZ method (Longa et al., 1989). All animals were fasted 12 h before surgery, and anesthetized with 3% isoflurane. The wound was cut about 2 cm in the middle of the neck, and the right common carotid artery, external carotid artery, and internal carotid artery were separated. After the common artery and external carotid artery, the internal carotid artery clipped. The common carotid artery was inserted into the wire plug, and the internal carotid artery was finally ligated, and the wound was sutured. After 90 min of ischemia, the plug was slowly withdrawn, and the blood reperfused into the left middle cerebral artery. The rats in the Sham group only separated the vascular arteries but did not ligature and thread.

### Drugs Injection

#### 5-Bromo-2′-Deoxyuridine Injection

The 5-bromo-2′-deoxyuridine (BrdU) (B5002, Sigma Co., Ltd., United States) powder dissolved in sterile physiological saline in the dark. Each group of experimental animals was injected intraperitoneally with the appropriate BrdU solution (50 mg/kg) once a day for 21 days before each EA treatment.

#### miR-146b Inhibitors Injection

The miR-146b inhibitor (GeneCopoeia Inc., Guangzhou China) diluted with 0.7% DMSO to a concentration of 10 μM at -20°C. Then 7 μl of the drug was administered to the left ventricle using a stereotaxic instrument 30 min before modeling (Zhan et al., 2010; Zhao et al., 2013). Simultaneously, 7 μl of 0.7% DMSO was injected into the MCAO group and the MCAO+EA group.

#### Scoring of Neurological Deficits

We performed neurobehavioral scoring and observed posture before and after EA treatment (modified Neurological Severity Scores, mNSS). The abnormality of the index was 0, the moderate abnormality was 1 point, and the severe abnormality was 2 points. The scores added together, and the total score is 0–18 points. The higher the score, the more serious the neuromotor injury.

#### Immunofluorescence

At the end of all experiments, rats anesthetized with sodium pentobarbital (800 mg/kg, i.p.). After perfusion, the brain tissue was fixed in 4% paraformaldehyde for 24 h and finally wrapped in paraffin after dehydration with gradient alcohol. The 5 μm thick brain slices were dewaxed and repaired for 15 min, and washed with PBS for 5 min. Then, 2N HCL was added dropwise for DNA denaturation, and the cells were incubated at 37°C for 1 h, washed three times with a boric acid solution (pH 8.5), then washed three times with PBS. The blocking solution was incubated at 37°C for 1 h, and then primary antibody BrdU (1:300; ab74547; Abcam, Cambridge), NeuN (1:200; ab177487; Abcam, Cambridge), DCX (1:100; ab18723; Abcam, Cambridge), NeuroD1 (1:200; ab60704; Abcam, Cambridge) were added dropwise. The next day, PBS washed three times, and the corresponding fluorescent secondary antibody was added dropwise in the dark. Nuclei of all cells counterstained with DAPI (1:1000; Santa Cruz, United States). The tissue slides was mounted in mounting medium (Vector Laboratories, United States), and images captured with a confocal fluorescence microscope (LSM710, Carl Zeiss, Germany). The average number of double-labeled positive cells from three slices for each rat with six fields of view/slice used for statistical analysis.

#### Western Blotting

The ischemic striatum were taken after the rats sacrificed, 1 ml of cell division fluid (Invitrogen; Thermo Fisher Scientific, Inc., United States) and 10 μl of PMSF storage solution added per 200 mg of brain tissue. It centrifuged, and the supernatant were taken to determine the concentration of protein. The protein samples were loaded to SDS-PAGE gel (Promega Corp., Madison, WI, United States) (20 V, l0 min, 60 V, 2 h), and then transferred to the PVDF membrane, 100 V, 60–120 min. Blocking for 2 h with 5% skim milk at room temperature, then incubation with HSP70 (1:1000; ab181606; Abcam, Cambridge), TSG101 (1:2000; ab125001; Abcam, Cambridge), CD81 (1:500; MA5-13548; Thermo Fisher Scientific, United States), NeuroD1 (1:1000; ab60704; Abcam, Cambridge), and β-action (1:8000; Proteintech, China) primary antibody at 4°C overnight, On the 2nd day, the corresponding secondary antibody goat anti-rabbit IgG (1:2000; ab6721; Abcam, Cambridge), Goat anti-mouse IgG (1:5000; ab6789; Abcam, Cambridge) were added and incubated for 2 h. Finally, the PVDF membrane were incubated with Enhanced Chemiluminescence (ECL) solution (RPN2232, GE Healthcare) and then placed on an image scanner for 1 min, and the expression levels of the target protein were analyzed by Image-Pro Plus software (version 7.0; UVP, LLC, Upland, CA, United States).

#### Exosome Isolation and miRNA Chip

Fresh frozen peri-ischemic striatum was dissected and treated with 20 units/ml papain (Worthington) in Hibernate E solution (2 ml/sample, BrainBits, Springfield, IL) for 15 min at 37°C. The tissue gently homogenized in 2 volumes (5 ml/sample) of cold Hibernate E solution. The tissue homogenate sequentially filtered through a 40 m mesh filter (BD Biosciences) and a 0.2 m syringe filter (Thermo Scientific). Exosomes were isolated from the filtrate as previously literature (Perez-Gonzalez et al., 2012). The filtrate was sequentially centrifuged at 300 g for 10 min at 4°C, 2,000 g for 10 min at 4°C, and 10,000 g for 30 min at 4°C to discard cells, membranes, and debris. Then, the exosomes were extracted according to the Exosome Isolation Kit (UR52121, Umibio, Shanghai, China). The total exosome RNA was isolated by the Exosome RNA isolation kit (Invitrogen), and microRNA expression profiling analyzed by the miRCURY LNA^TM^ microRNA array system (Exiqon).

#### Real-Time qRT-PCR

Total RNA was extracted from the peri-ischemic striatum using TRIZOL reagent (Invitrogen, Thermo Fisher Scientific, Inc., United States). Reverse transcription reaction and PCR amplification reaction carried out using the miRNA All-in-one^TM^ qRT-PCR miRNA Detection kit, and amplification performed on an ABI7500 real-time PCR instrument, and the results were normalized to GAPDH gene expression. All experiments were performed in triplicate and repeated at least three times. The following primer sequences used: miR-146b (forward) 5’-UGAGAACUGAAUUCCAUAGGCUGU-3’, (reverse) 5’-ACUCUUGACUUAAGGUAUCC GACA-3’. GAPDH (forward) 5’-AGACAGCCGCATCTTCTTGT-3’, (reverse) 5’-CTT GCCGT GGGTAGAGTCAT.

#### Oxygen-Glucose Deprivation and Reperfusion

Neural stem cells were isolated from E13.5 to E14.5 rat embryonic brains. The dissected cerebral tissues were cut into small chunks and were digested with Accutase solution (A6964, Sigma-Aldrich, Millipore, United States) for 5 min at 37°C and washed with PBS solution, plated in a six-well plastic plate in Neurobasal medium (Gibco, United States) supplemented with 20 ng/ml basic fibroblast growth factor (Gibco, United States), 20 ng/ml epidermal growth factor (Gibco, United States), 2% B27 supplement (Gibco, United States), and 1% penicillin/streptomycin, and incubated at 37°C in 5% CO_2_.

To mimic the condition of ischemic injury *in vitro*, the neural stem cells were cultured in oxygen-glucose deprivation (OGD) medium with DMEM, then placed in a modular chamber (MC-101 model, Billups-Rothenberg, Del Mar, CA) filled with a gas mixture (1% O_2_, 5% CO_2_, and 94% N_2_) for 6 h. Then, by changing the culture medium for OGD reperfusion (OGD/R), the neural stem cells were restored to a normoxic state for 72 h. Control group of neural stem cells cultured under standard conditions without any treatment.

#### Cell Model and Experimental Group

This study was divided into three groups, Control group, OGD/R group, OGD/R+miR-146b inhibitors group. Control group: primary neural stem cells were normally cultured; OGD/R group: primary neural stem cells were cultured with OGD 6 h and reperfusion 72 h; OGD/R+miR-146b inhibitors group: neural stem cell were cultured with adding 7 μl 10μM miR-146b inhibitors on OGD 6 h and reperfusion 72 h.

#### Statistical Analysis

All quantitative data are expressed as the means ± *SD* and were analyzed using SPSS 21.0 statistical analysis software (IBM, Corp., Armonk, NY, United States). One-way ANOVA analyzed the mNSS scores, western blotting, qRT-PCR, and immunofluorescence staining results. *P*< 0.05 was considered to indicate a statistically significant difference.

## Results

### EA Enhanced the Expression of Exosomal miR-146b in Peri-Ischemic Striatum

**FIGURE 1:**
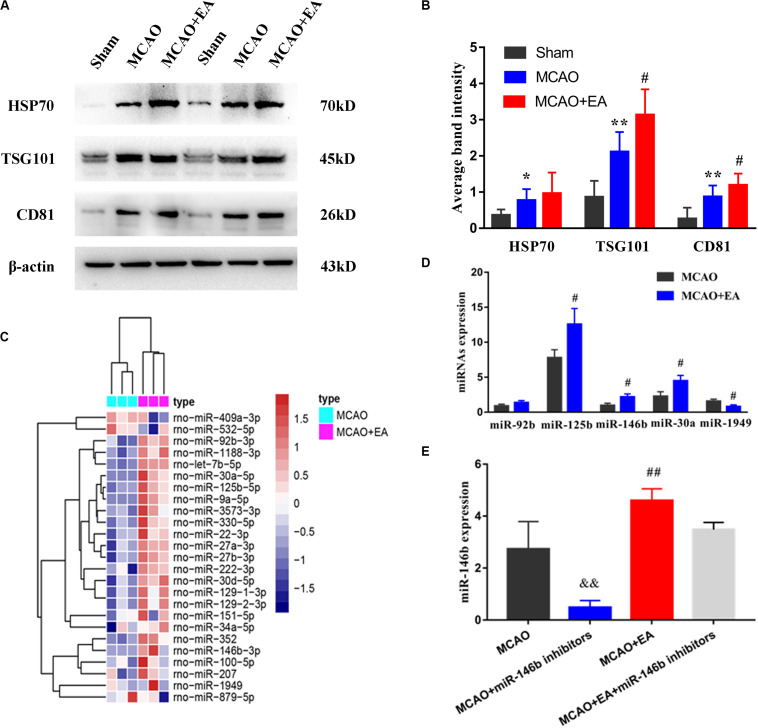
Electro-acupuncture promotes the release of exosomal miR-146b in peri-ischemic striatum. **(A)** The expression of exosome-associated markers HSP70, TSG101, and CD81 were analyzed by Western blotting. **(B)** The expression of markers were quantified on the basis of average band intensity (*n* = 4). **(C)** MiRNA microarray analysis of exosomes from peri-ischemic striatum and clustering of 25 significantly altered miRNAs with ≥1.5-fold difference in expression between MCAO and MCAO+EA groups. **(D)** Four miRNAs were significantly upregulated and 1 miRNA were downregulated in exosomes of the MCAO+EA group compared with the MCAO group were checked by RT-qPCR (*n* = 3). **(E)** The expression of miR-146b in tissues of peri-ischemic striatum were analyzed form MCAO, MCAO+miR-146b inhibitors, MCAO+EA, MCAO+EA+miR-146b inhibitors groups (*n* = 3). Data represent means ± *SD* of the results of representative experiment. *P< 0.05, MCAO vs. Sham; **P < 0.01, MCAO vs. Sham; ^#^*P*< 0.05 and ^##^*P*< 0.01, MCAO vs. MCAO+EA; ^&&^*P*< 0.01, MCAO vs. MCAO+miR-146b inhibitors; MCAO+EA vs. MCAO+EA+miR-146b inhibitors.

Firstly, we found that the expression of HSP70, TSG101, and CD81 of exosomal biomarkers were increased in the MCAO group compared with the Sham group. However, after EA treatment, the expression of CD81 and TSG101 were increased in the MCAO+EA group compared with the MCAO group (*P*< 0.05, Figures 1A,B).

Then, exosomes were isolated from peri-ischemic striatum of the MCAO group and the MCAO+EA group, and furtherly analyzed miRNAs expressed changes in these exosomal by microarray chip, using a 1.5-fold change and *P*< 0.05 as the threshold cutoff for screening differentially expressed miRNAs. We found that 25 miRNAs differentially expressed in exosomes, including miR-409a, miR-532, miR-1188, miR-125b, miR-146b, miR-30a, etc. (Figure 1C). Furthermore, the expression of three miRNAs (miR-125b, miR-146b, miR-30a) were upregulated, and miR-1949 was downregulated by real-time PCR, consistent with chip results (*P <* 0.05, Figure 1D), and miR-92b was not statistically significant but had an upward trend (*P >* 0.05, Figure 1D). Due to miR-146b has been proved to be involved in the differentiation of neural stem cells (Xiao et al., 2015) we explored whether EA regulated the miR-146b expression in peri-ischemic striatum. The results showed that the expression of miR-146b was decreased in the MCAO+miR-146b inhibitors group, while higher expression in the MCAO+EA group, which compared with the MCAO group (*P <* 0.01, Figure 1E). Moreover, it had lower expression in the MCAO+EA+miR-146b inhibitors group compared with the MCAO+EA group, but no statistical significance (*P >* 0.05, Figure 1E). These results suggest that EA is involved in regulating the expression of exosomal miR-146b in peri-ischemic striatum.

### EA Promoted the Differentiation of Endogenous Neural Stem Cells via miR-146b in Peri-Ischemic Striatum and SVZ of the Ischemic Hemisphere

**FIGURE 2:**
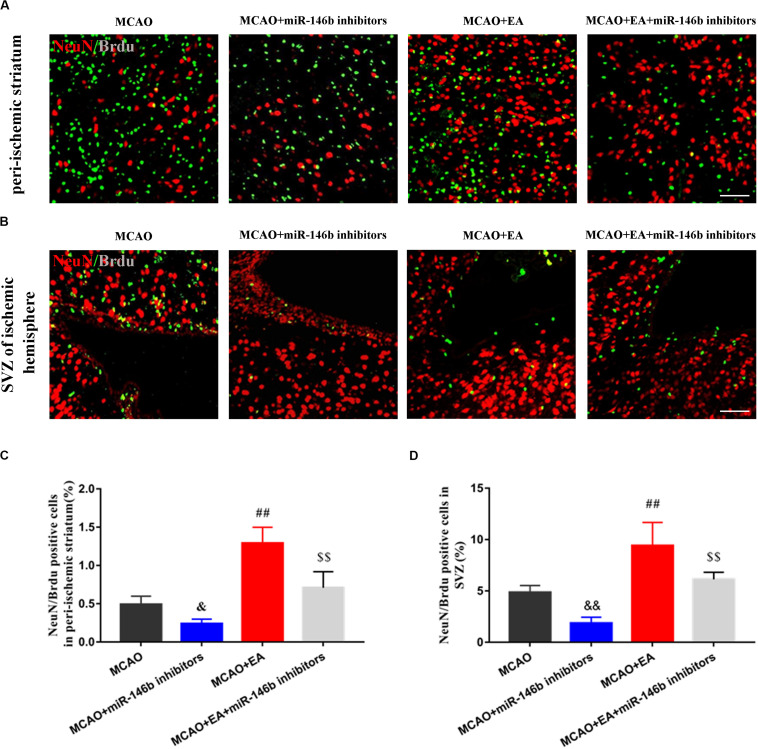
Electro-acupuncture promotes the differentiation of endogenous neural stem cells into neurons in peri-ischemic striatum and SVZ of ischemic hemisphere. **(A)** Immunofluorescence and co-localization of NeuN with BrdU in peri-ischemic striatum **(B)**. Immunofluorescence and co-localization of NeuN with BrdU in the subventricular zone (SVZ) of ischemic hemisphere. **(C,D)** The positive cells of co-localization of NeuN/Brdu in peri-ischemic striatum and SVZ of ischemic hemisphere, respectively (*n* = 4). Data represent means ± *SD* of the results of representative experiment. ^&^*P*< 0.05 and ^&&^*P*< 0.01, MCAO vs. MCAO+miR-146b inhibitors; ^##^*P*< 0.01, MCAO vs. MCAO+EA; ^$$^*P*< 0.05, MCAO+miR-146b inhibitors vs. MCAO+EA+miR-146b inhibitors.

To explore whether EA regulated the differentiation of neural stem cells through miR-146b, we used BrdU to label neonatal cells and NeuN to mark neural cells after ischemic stroke. Thus immunofluorescence co-localization with NeuN and BrdU represented neonatal neurons. In peri-ischemic striatum and SVZ of the ischemic hemisphere, we found that the positive cells of co-localization of NeuN^+^ /Brdu^+^ were decreased in the MCAO+miR-146b inhibitors group and increased in the MCAO+EA group, compared with the MCAO group (*P*< 0.05 or *P*< 0.01, Figures 2A–D). Besides, compared with the MCAO+EA group, the result also showed that the co-localization of the MCAO+EA+miR-146b inhibitors group was decreased (*P*< 0.01, Figures 2A–D). It indicated that EA could promote endogenous neural stem cells differentiation into neurons in peri-ischemic striatum and SVZ of the ischemic hemisphere through miR-146b after ischemic stroke.

### MiR-146b Regulated the Differentiation of Neural Stem Cells *in vitro* OGD/R Injury

**FIGURE 3:**
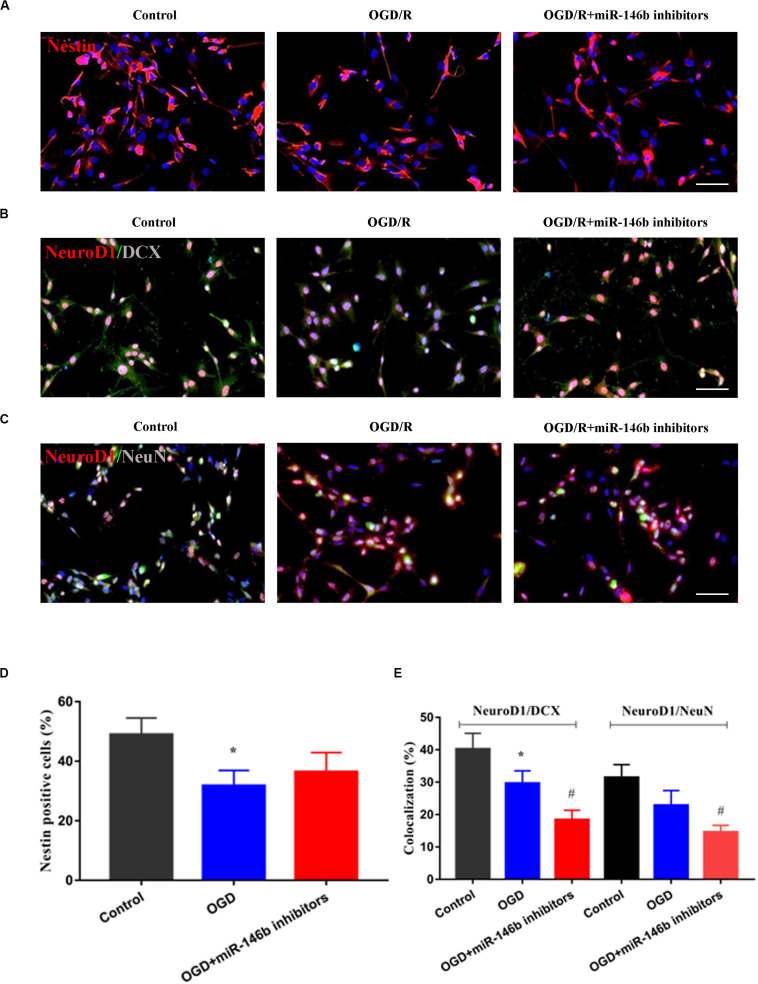
MiR-146b inhibitors suppressed neuronal differentiation of stem cells on OGD 6 h and reperfusion 72 h of *vitro* culture. **(A)** Cells were immunostained for Nestin (red) and DAPI for the nucleus (blue). **(B)** Immunofluorescence and co-localization of NeuroD1 (red) with DCX (green) (*n* = 3). **(C)** Immunofluorescence and co-localization of NeuroD1 (red) with NeuN (green). **(D)** Percentage of Nestin positive cells in control, OGD/R, OGD/R+miR-146b inhibitors groups (*n* = 3). **(E)** Percentage of co-localization with NeuroD1^+^/DCX^+^ and NeuroD1^+^/NeuN^+^ in control, OGD/R, OGD/R+miR-146b inhibitors groups (*n* = 3). Data represent means ± *SD* of the results of representative experiment. *P< 0.05, Control vs. OGD/R; ^#^*P*< 0.05, OGD/R vs. MCAO+miR-146b inhibitors.

Furthermore, we performed neural stem cells OGD/R culture to mimic *in vitro* ischemic injury model. The results showed that OGD/R induced Nestin-marked neural stem cell injury, and miR-146b inhibitors could suppress the injury, but no statistical significance (*P >* 0.05, Figures 3A,D). Simultaneously, OGD/R inhibited the differentiation of neural stem cells, and miR-146b inhibitors further suppressed the differentiation of neural stem cells and NeuroD1 was involved in neural stem cells differentiation into neurons with NeuroD1^+^/DCX^+^ and NeuroD1^+^/NeuN^+^ (*P <* 0.05, Figures 3B–E). These results suggest that miR-146b could regulate NeuroD1-mediated neural stem cells differentiation *in vitro* OGD/R injury.

### EA Could Promote NeuroD1-Mediated Neural Stem Cells Differentiation via miR-146b in SVZ of the Ischemic Hemisphere

**FIGURE 4:**
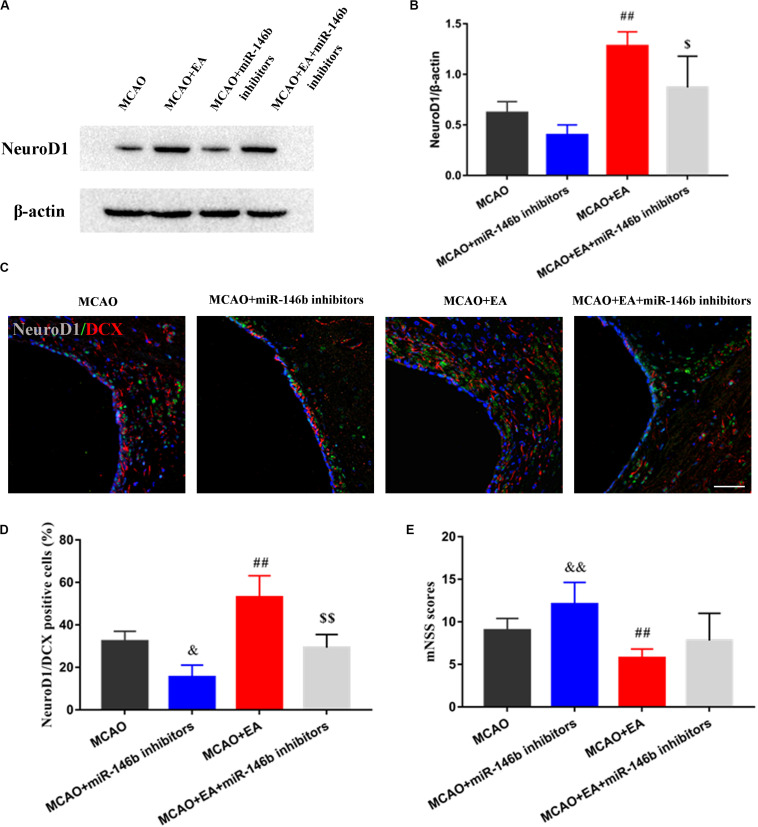
Electro-acupuncture promotes NeuroD1 associated neuron differentiation in SVZ of ischemic hemisphere. **(A)** The expression of NeuroD1 of neuronal differentiation-associated marker was analyzed by Western blotting in SVZ of ischemic hemisphere. **(B)** The expression of markers were quantified on the basis of average band intensity (*n* = 3). **(C)** Immunofluorescence and co-localization of NeuroD1 with DCX in SVZ of ischemic hemisphere. **(D)** Co-localization of NeuroD1 with DCX positive cells (*n* = 3). **(E)** The mNSS scores were tested in MCAO, MCAO+miR-146b inhibitors, MCAO+EA, MCAO+EA+miR-146b inhibitors groups (*n* = 10). Data represent means ± *SD* of the results of representative experiment. ^##^*P*< 0.01, MCAO vs. MCAO+EA; ^&^*P*< 0.05 and ^&&^*P*< 0.05, MCAO vs. MCAO+miR-146b inhibitors; ^$^*P*< 0.05 and ^$$^*P*< 0.05, MCAO+EA vs. MCAO+EA+miR-146b inhibitors.

To furtherly clarify whether EA promoted the differentiation of neural stem cells through miR-146b, firstly, the expression of NeuroD1 of differentiation-associated factors in SVZ of the ischemic hemisphere with miR-146b inhibitors intervention was observed, and the results showed that the expression of NeuroD1 in the MCAO+miR-146b inhibitors group was lower trend than that in the MCAO group, and it was significantly increased after EA in SVZ of the ischemic hemisphere (*P <* 0.01, Figures 4A,B). Moreover, it had lower expression in the MCAO+EA+miR-146b inhibitors group compared with the MCAO+EA group (*P <* 0.05, Figures 4A,B). Subsequently, we used immunofluorescence staining to co-localize NeuroD1 and DCX in SVZ of the ischemic hemisphere. Unsurprisingly, more NeuroD1^+^/DCX^+^ were co-localized in the MCAO+EA group, and injected miR-146b inhibitors could decrease the number of NeuroD1^+^/DCX^+^ co-localization (*P <* 0.05, Figures 4C,D).

Furthermore, we assessed the neurological deficits, the results showed that the mNSS score was obviously reduced in the MCAO+EA group compared with the MCAO group, and it was reduced in the MCAO+EA+miR-146b inhibitors group compared with MCAO+miR-146b inhibitor group as well (*P <* 0.05, Figure 4E), which proved that EA could improve neurological deficits after stroke.

## Discussion

Stroke is considered the leading cause of neurological dysfunction and death worldwide. Currently, recombinant tissue plasminogen activator (rt-PA) is the only thrombolytic drug approved by the FDA for the treatment of ischemic stroke. However, due to the time window limitation of thrombolytic therapy and serious adverse prognosis, its clinical application is greatly limited (Powers et al., 2019). Non-drug treatment of stroke is more and more common in clinic. EA has been shown to effectively improve neurological deficits after stroke (Chang et al., 2017). In this study, we found that the content of exosomes was increased in the peri-ischemic striatum after EA. Then, we revealed 25 differentially expressed miRNAs in exosomes by analysis of miRNA expression profile, of which miR-146b was selected for further analysis, by injecting miR-146b inhibitor into the lateral ventricle of rats, it has been determined that miR-146b was involved in the regulation of EA on neurogenesis. Subsequently, we demonstrated that EA could promote endogenous neural stem cells differentiation into neurons in peri-ischemic striatum and SVZ of the ischemic hemisphere by up-regulating the expression of miR-146b after ischemic stroke. *In vitro* and *in vivo* we verified the role of miR-146b on neurogenesis, which proved that miR-146b was involved in promoting NeuroD1-mediated neural stem cells differentiation. In addition, EA also could improve neurological deficits through miR-146b after ischemic stroke. Therefore, we speculated that exosomal miR-146b might be a potential target for promoting endogenous neural stem cells differentiation after stroke.

Neurogenesis provides a potential therapeutic method for stroke. In an experimental stroke, cerebral ischemic neurogenesis was increased in ipsilateral SVZ, and neuroblasts migrate from SVZ to the ischemic border region of the striatum and cortex, which have the phenotype of mature neurons (Liu et al., 2007). In recent years, studies have shown that exosomes mediated delivery of proteins, lipids, and nucleic acids, which are involved in neurogenesis (Lässer, 2012). These bioactive molecules mediate exosomal cell-to-cell communication which may target specific cell types. Exosomes released by the neural cells not only regulate the occurrence and development of nervous system diseases, but also play a role in regeneration and remodeling of the nervous system after neural injury (Porro et al., 2015). It reported that exosome treatment significantly restore cerebral infarct volume and improve neurological deficits in cerebral ischemia rats (Zheng et al., 2019). In a study, the researchers co-cultured the damaged cortical neurons with adipose stem cells, and found that exosomes secreted by neural stem cells can exert neuroprotective functions by inhibiting neuronal apoptosis, thereby promoting the central nervous system (CNS) regeneration and repair (Hong et al., 2019). Another study showed that mesenchymal stem cell-derived exosomes stimulated neurogenesis in the SVZ region and reduced neurological damage (Reza-Zaldivar et al., 2019). In this study, we found that the content of exosomes was increased in the peri-ischemic striatum after EA treatment, accompanied by the improvement of neurological deficits after stroke.

Stem cells can self-renew or differentiate into multiple cell types, and miRNAs play a central role in determining the fate of stem cells. Thus, does exosomes play an important role by delivering miRNAs? We revealed that exosomal miR-146b identified by microarray chip influenced the differentiation of endogenous neural stem cells. Previous study has shown that miR-146 regulates differentiation of neural stem cells by regulating Notch 1 signaling pathway (Mei et al., 2011). *In vitro* experiment, human neural stem cells (hNSCs) were induced to differentiation with 2D and 3D culture systems, and using miRNA PCR arrays to identify differential expression profiles in differentiated hNSCs, revealing hsa-miR-146b was upregulation, and it further was confirmed that miR-146b regulated the differentiation of hNSCs into neurons by its mimic transfection (Xiao et al., 2015).

Increasing evidence suggests that miRNAs are involved in the process of ischemic stroke (Zhang et al., 2017). A large number of studies have used miRNA treatment to reduce ischemic brain injury and functional recovery after stroke (Volný et al., 2015; Sen, 2019; Van Kralingen et al., 2019; Yuan et al., 2019). Based on this fact, we explored the effect of exosomal miR-146b on neural stem cell differentiation after ischemia-reperfusion injury. We demonstrated that EA could promote endogenous neural stem cells differentiation into neurons in peri-ischemic striatum and SVZ of the ischemic hemisphere via the upregulation of miR-146b after ischemic stroke. Besides, *in vitro* ischemic injury model, we found that OGD/R could inhibit the differentiation of neural stem cells, and miR-146b inhibitors further suppressed the differentiation of neural stem cells. We also confirmed that NeuroD1 was involved in neural stem cells differentiation into neurons. *In vitro* and *in vivo*, we verified the effect of miR-146b on neurogenesis, which proved that miR-146b was involved in promoting NeuroD1-mediated neural stem cells differentiation.

As a neurodifferentiation factor, NeuroD1 is essential for neuronal development, and it can reprogram other cell types into neurons (Pataskar et al., 2016). In our previous study, it has shown that EA could regulate the Notch1 signaling pathway (Tao et al., 2015). While the Notch1 signal transduction may be due to its inhibitory effect on bHLH protein that activates the cell differentiation program (Kageyama et al., 2005). As a type of bHLH protein, NeuroD1 is a potential downstream target of the Notch signaling pathway. Studies have shown that Notch signaling increased, and the transcription level of NeuroD1 was decreased (Fineberg et al., 2012). Therefore, we speculated that NeuroD1 might be a potential factor for EA to promote the differentiation of endogenous neural stem cells. In our study, EA could promote neurogenesis after stroke and increase the number of DCX^+^/NeuroD1^+^ colocalization in the SVZ. And when we injected miR-146b inhibitor, the number of DCX^+^/NeuroD1^+^ co-localization was decreased. Therefore, our results suggest that NeuroD1 might be a downstream factor of exosomal miR-146b, which could promote the differentiation of endogenous neural stem cells in rats with ischemia-reperfusion injury. However, in the future, we will further explore how mir-146b regulates NeuroD1-mediated differentiation of neural stem cells.

## Conclusion

Electro-acupuncture is a prospective therapy that can enhance the differentiation of endogenous neurogenesis and improve neurological deficits after ischemic stroke. We demonstrated that exosomal miR-146b is an important neuromodulator of neurogenesis, which promotes endogenous neural stem cell differentiation into neurons in peri-ischemia after stroke.

## Data Availability Statement

The raw data supporting the conclusions of this article will be made available by the authors, without undue reservation, to any qualified researcher.

## Ethics Statement

The animal study was reviewed and approved by the Animal Ethics Committee of Fujian University of Traditional Chinese Medicine (SCXK#2016005).

## Author Contributions

LC, WL, and JT contributed to the study concept and design. SZ, TJ, LW, YuZ, YiZ, YL, and XH collected and analyzed the data. HL and MY performed the statistical analyses. WL and JT drafted and revised the manuscript. All authors contributed to the critical revision of the manuscript, approved the final manuscript as submitted, and agreed to be accountable for all aspects of the work.

## Conflict of Interest

The authors declare that the research was conducted in the absence of any commercial or financial relationships that could be construed as a potential conflict of interest.
