# Supplementary material for: Electro-Acupuncture Promotes the Differentiation of Endogenous Neural Stem Cells via Exosomal microRNA 146b After Ischemic Stroke
Source: Front Cell Neurosci. 2020 Jul 21;14:223. doi: 10.3389/fncel.2020.00223 (PMC7385414; doi:10.3389/fncel.2020.00223)
Supplement: Supplementary file 1 [file Data_Sheet_1.PDF]

MiRNA microarray data：

Name	MCAO3	MCAO6	MCAO1	MCAO+EA 3	MCAO+EA 8	MCAO+EA 5
rno-miR-1188-3p	0.15789474	0.18090452	0.184327	0.238489	0.285947	0.273828
rno-miR-129-1-3p/ 129-2-3p	1.27406354	0.75488554	1.054786	1.455726	2.189908	2.364095
rno-miR-3573-3p	0.55002371	0.49860413	0.429912	0.886659	0.677699	1.077156
rno-let-7b-5p	7.45092461	7.7414852	7.765453	11.96694	11.92838	12.22289
rno-miR-100-5p	1.79468943	1.60692351	1.375828	1.874026	1.567553	2.460414
rno-miR-125b-5p	25.865339	20.824679	26.20806	50.21783	43.00054	61.74887
rno-miR-151-5p	0.56187767	0.31658292	0.628587	0.288076	0.763429	0.962683
rno-miR-1949	0.21906117	0.25181463	0.200331	0.361865	0.195334	0.20474
rno-miR-207	2.70886676	3.84924623	2.857064	3.854782	3.243625	4.480585
rno-miR-222-3p	5.94167852	4.88553881	3.64128	6.903778	6.607162	7.336359
rno-miR-22-3p	13.0972025	7.31379118	13.76711	18.5608	19.34075	27.51286
rno-miR-27a-3p	3.33997155	2.06588498	2.831126	4.557261	4.499186	5.055472
rno-miR-27b-3p	4.08250356	3.15745394	3.878035	5.283943	4.875746	5.690872
rno-miR-30a-5p	8.52916074	8.59184813	8.481788	11.20189	10.4943	12.87948
rno-miR-30d-5p	3.50213371	2.29034059	2.972406	4.446281	5.384699	4.960161
rno-miR-330-5p	0.38359412	0.28592965	0.315121	0.520425	0.485621	0.723147
rno-miR-34a-5p	2.41394026	0.62255723	1.689845	2.319362	2.933261	2.159355
rno-miR-352	3.21716453	3.39642658	3.628587	5.237308	4.291373	5.349975
rno-miR-377-3p	0.06543386	N/A	0.05298	N/A	0.049919	0.051437
rno-miR-146b-3p	0.72261735	0.69530988	0.70585	1.316411	0.782963	1.139183
rno-miR-409a-3p	0.056899	0.0731435	0.066777	0.015939	0.0293	0.065557
rno-miR-532-5p	0.09056425	0.11781128	0.092715	0.038371	0.095496	0.065053
rno-miR-879-5p	0.14651494	0.13735343	0.201987	0.154664	0.091698	0.147907
rno-miR-92b-3p	0.06875296	0.08654383	0.070088	0.100354	0.108058	0.10943
rno-miR-9a-5p	14.6770982	14.0820771	14.63521	25.17237	21.63592	29.03833
