# Supplementary material for: Electro-Acupuncture Promotes the Differentiation of Endogenous Neural Stem Cells via Exosomal microRNA 146b After Ischemic Stroke
Source: Front Cell Neurosci. 2020 Jul 21;14:223. doi: 10.3389/fncel.2020.00223 (PMC7385414; doi:10.3389/fncel.2020.00223)
Supplement: Supplementary file 2 [file Data_Sheet_2.PDF]

The expression of exosome-associated markers HSP70, TSG101 and CD81 were analyzed by Western blotting.

Sham MCAO MCAO+EA Sham MCAO MCAO+EA

HSP70

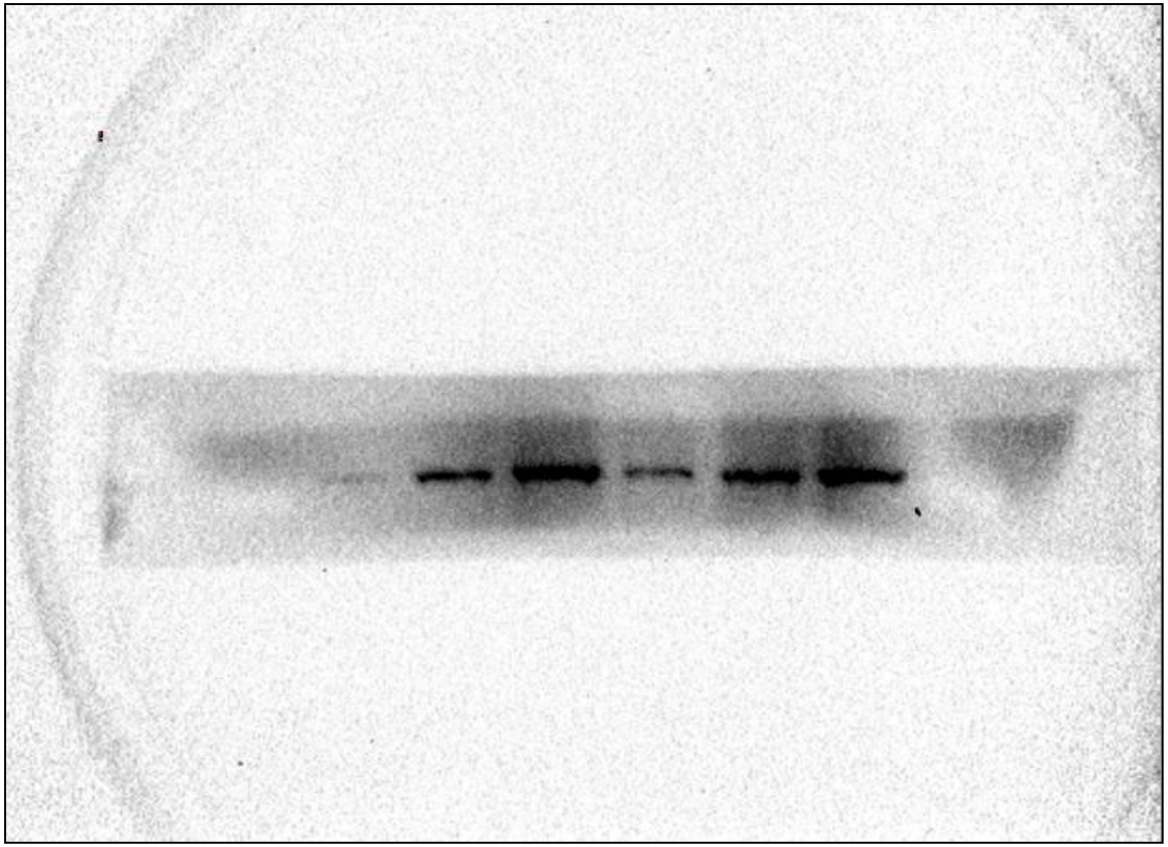

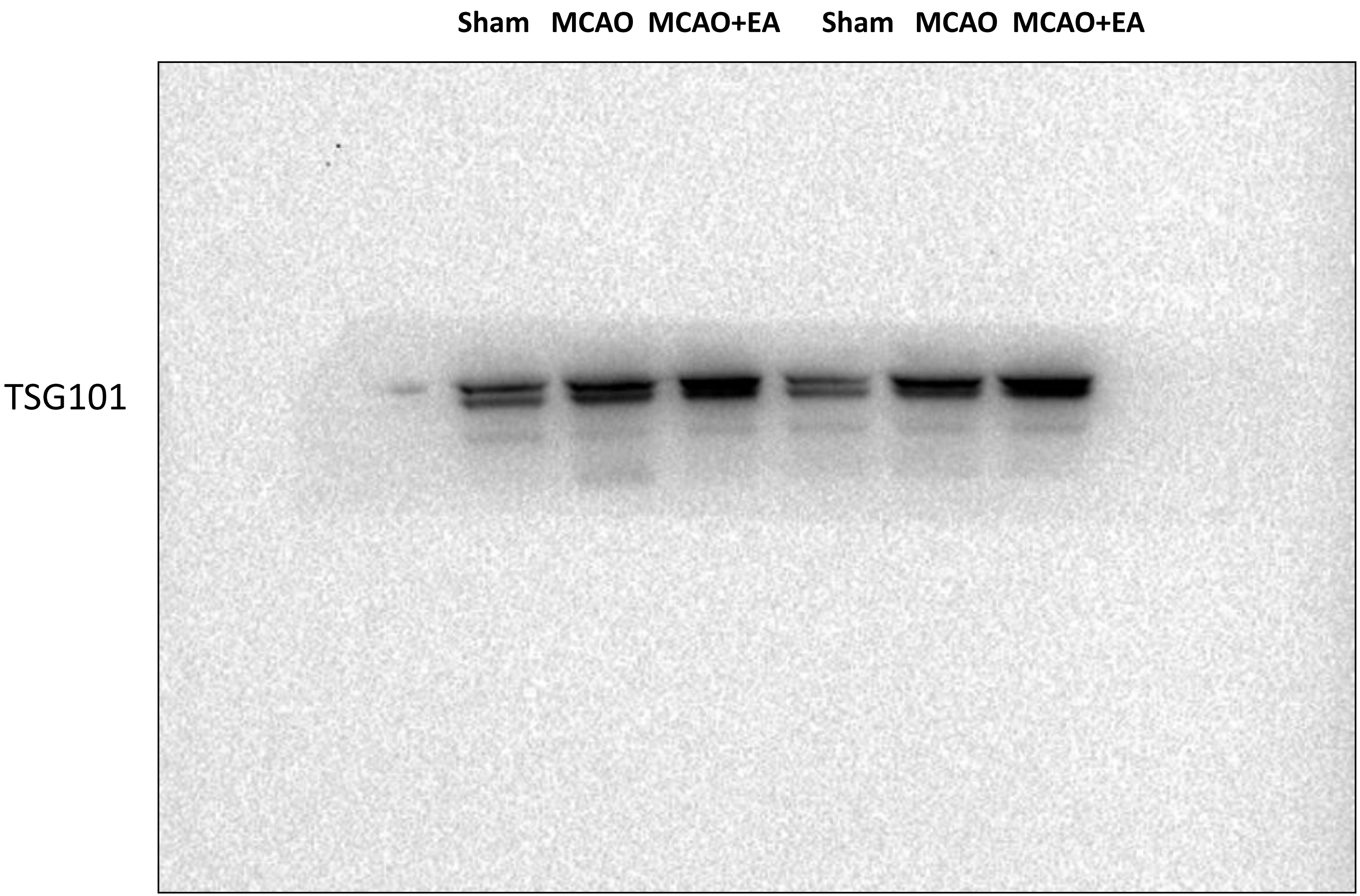
Sham
MCAO
MCAO+EA
Sham
MCAO
MCAO+EA
TSG101

Sham MCAO MCAO+EA Sham MCAO MCAO+EA

CD81

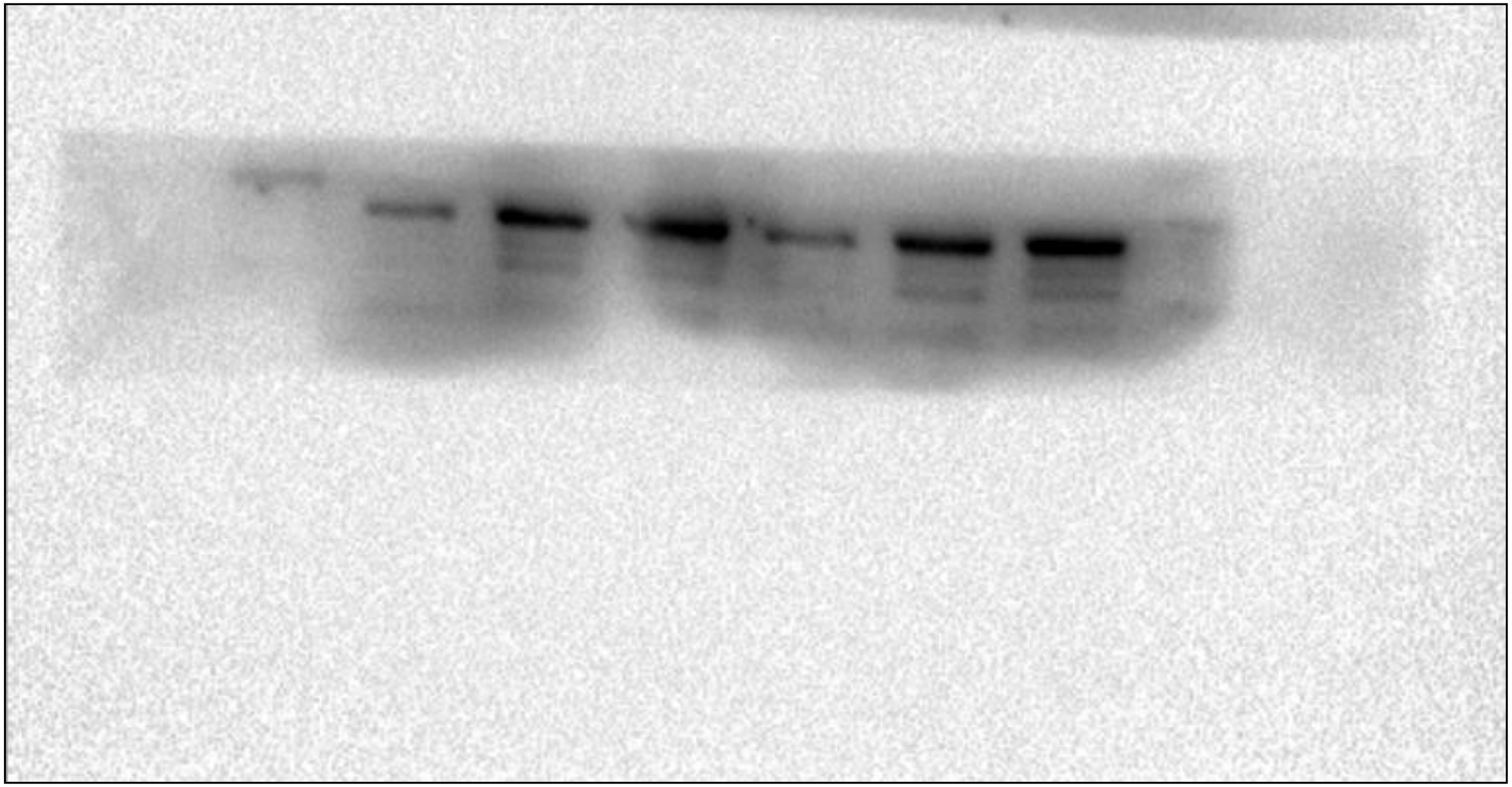

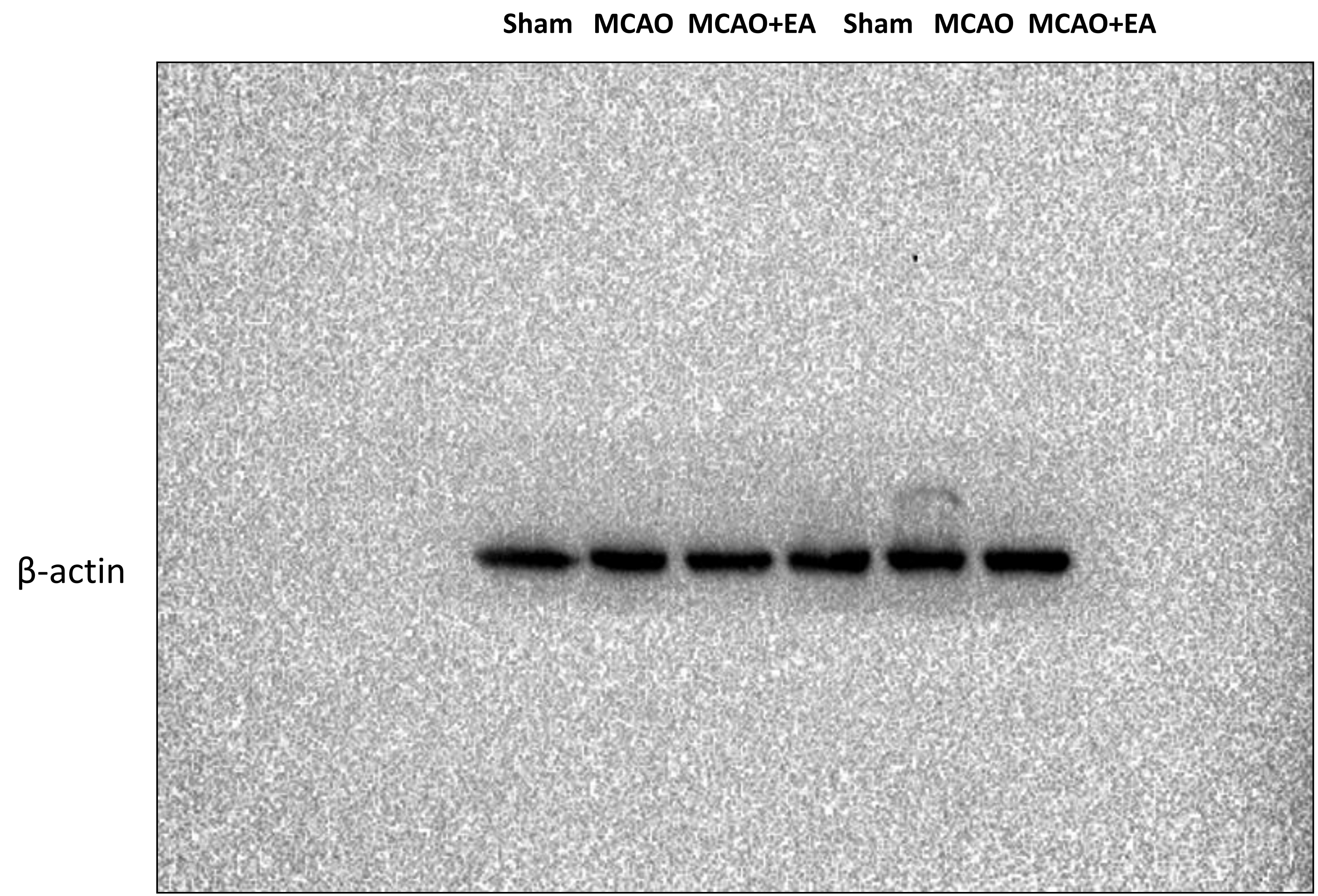
Sham MCAO MCAO+EA Sham MCAO MCAO+EA
β-actin

The expression of neuron differentiation-associated marker NeuroD1 were analyzed by Western blotting.

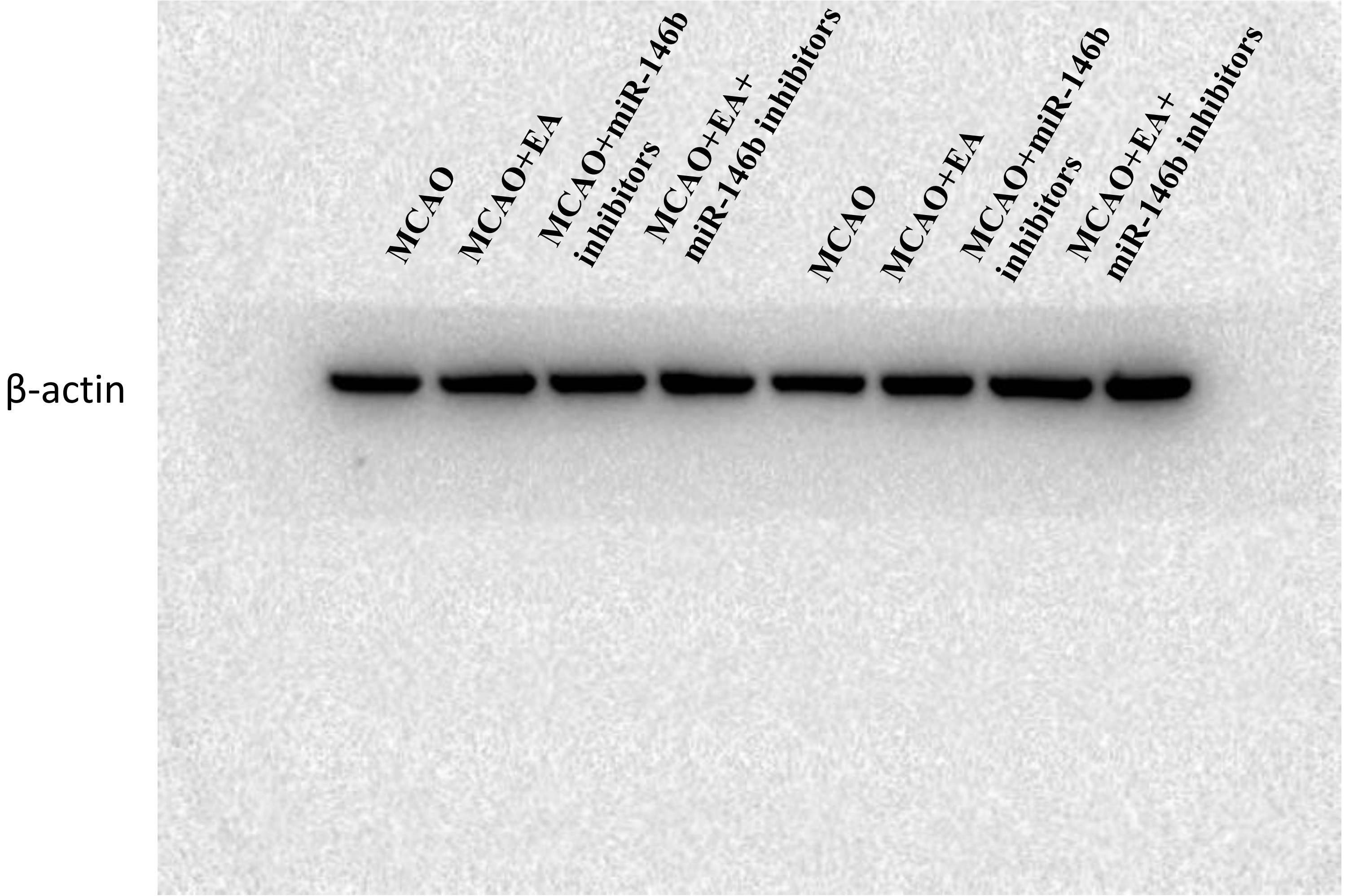

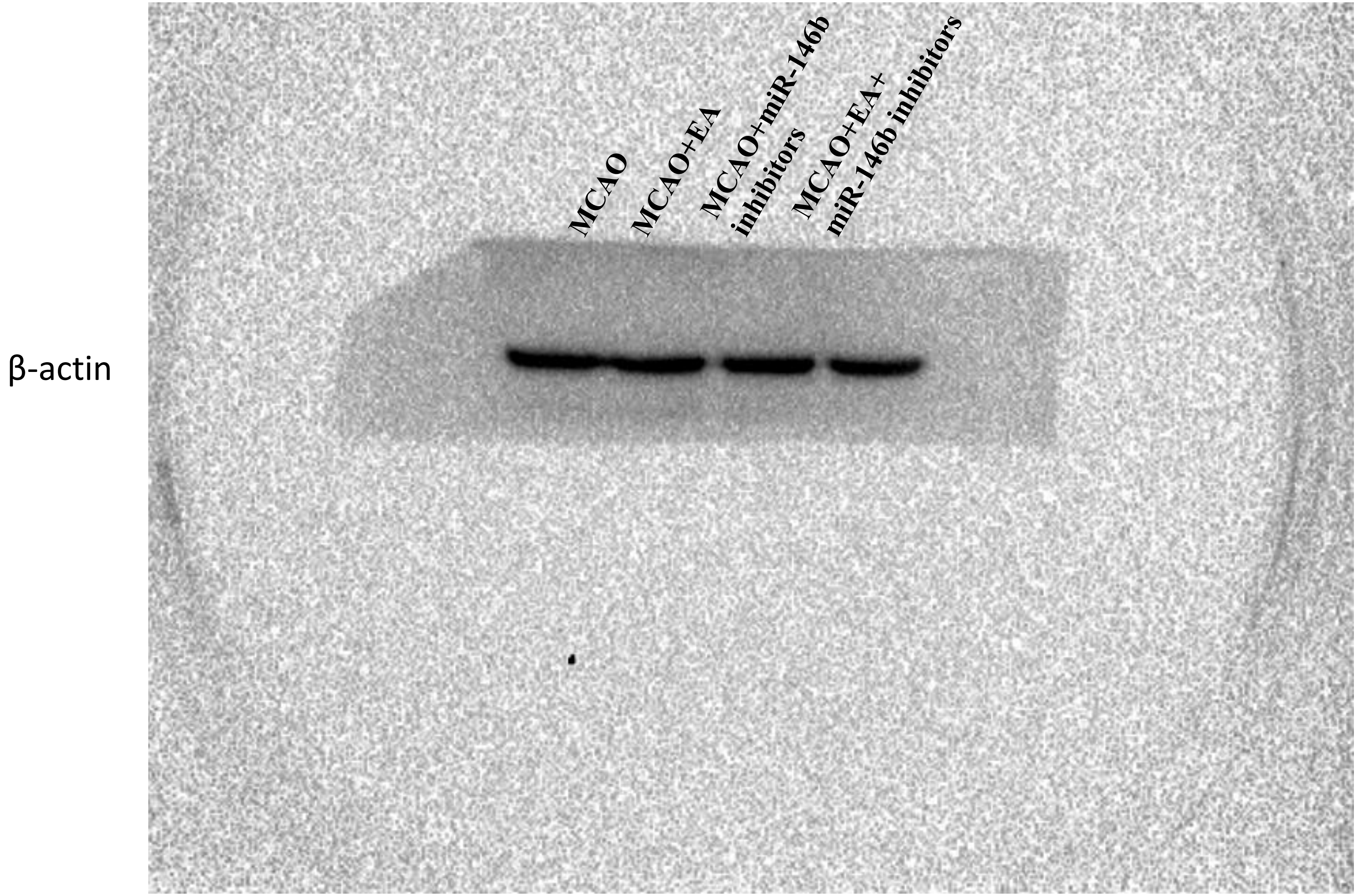
β-actin
MCAO
MCAO+EA
MCAO+miR-146b inhibitors
MCAO+EA+ miR-146b inhibitors

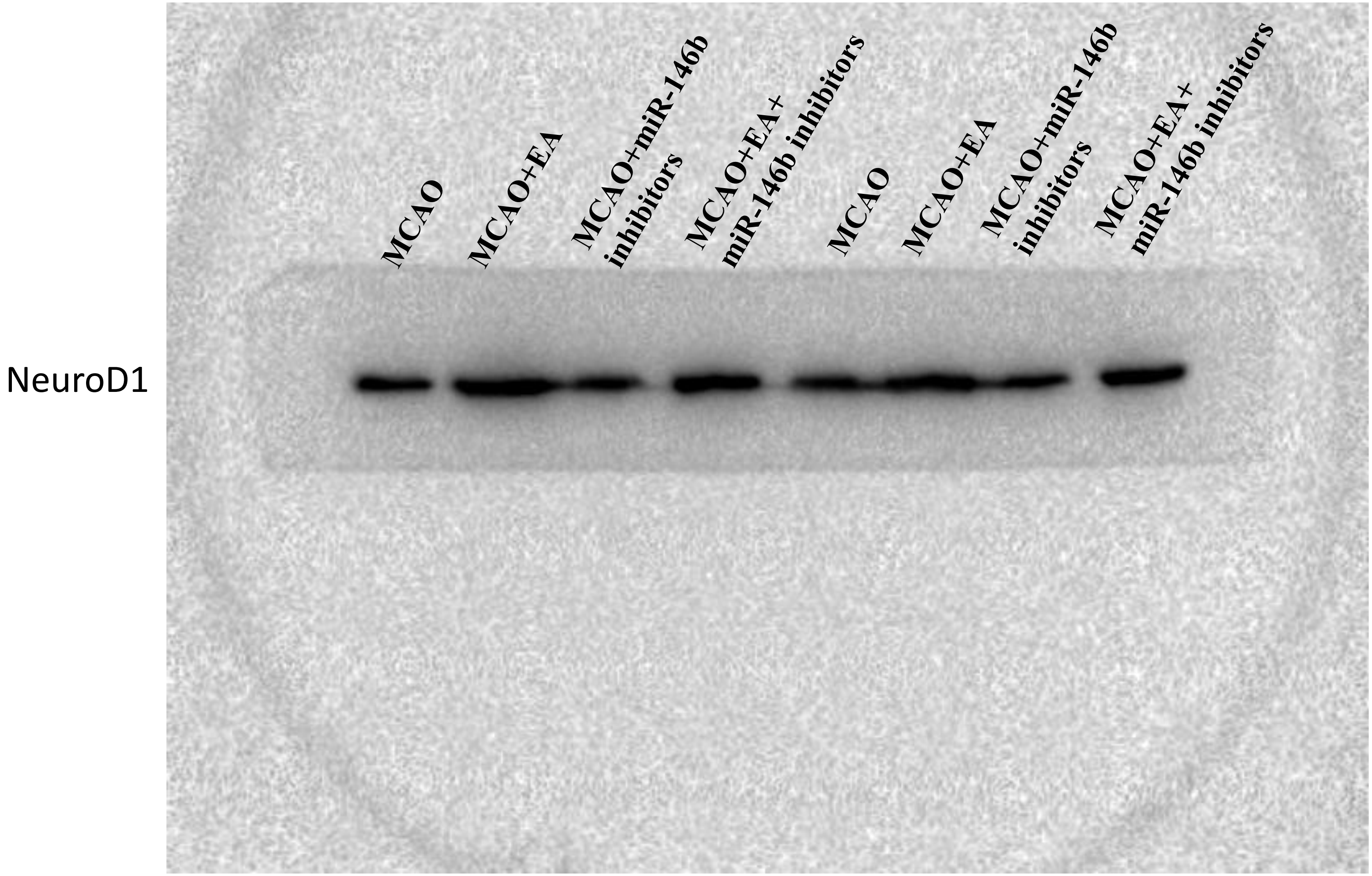

MCAO
MCAO+EA
MCAO+miR-146b inhibitors
MCAO+EA+ miR-146b inhibitors
MCAO
MCAO+EA
MCAO+miR-146b inhibitors
MCAO+EA+ miR-146b inhibitors
NeuroD1

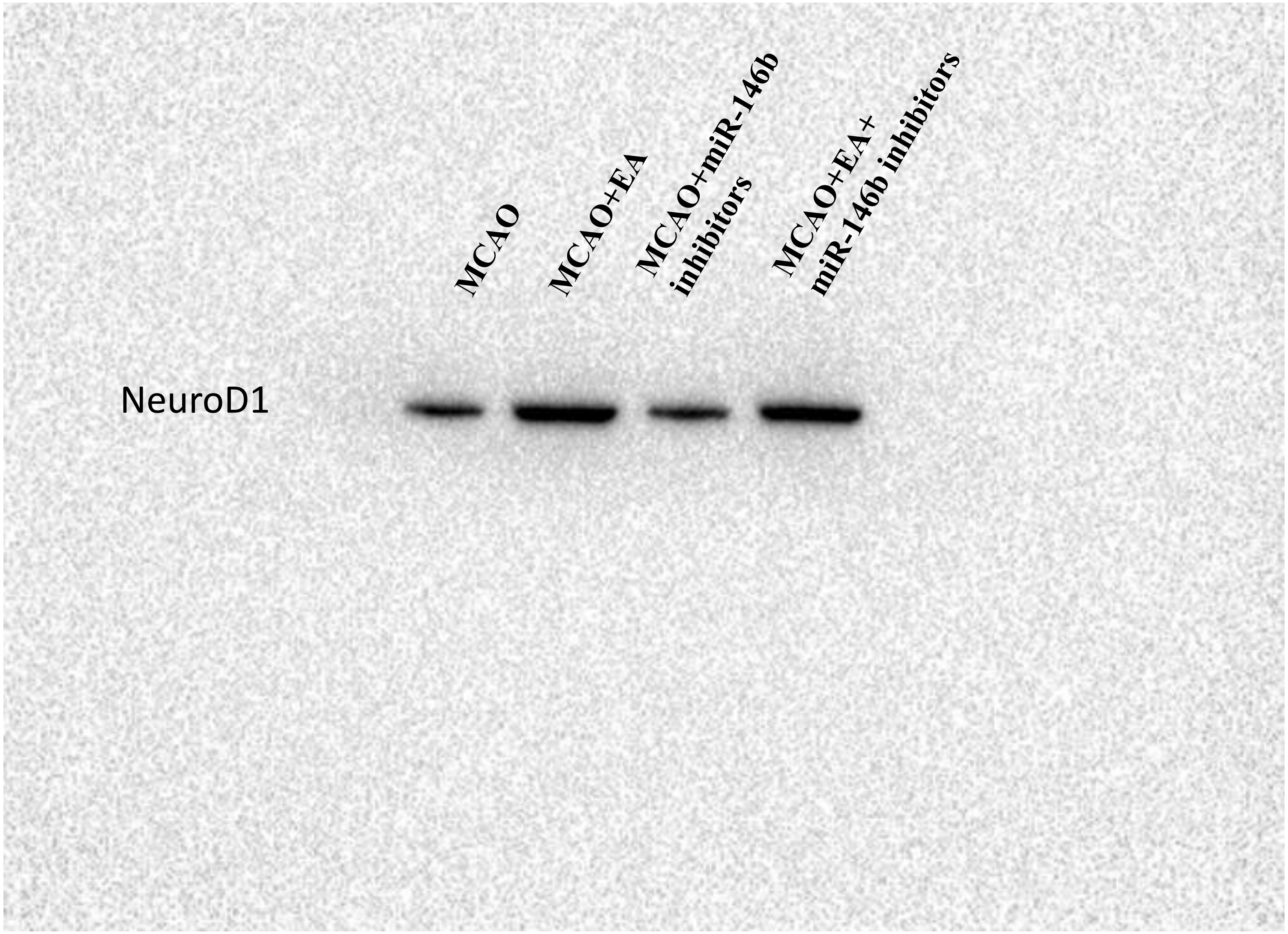

MCAO
MCAO+EA
MCAO+miR-146b inhibitors
MCAO+EA+ miR-146b inhibitors
NeuroD1
